# Perceived Neighborhood Environmental Attributes Associated with Walking and Cycling for Transport among Adult Residents of 17 Cities in 12 Countries: The IPEN Study

**DOI:** 10.1289/ehp.1409466

**Published:** 2015-07-17

**Authors:** Jacqueline Kerr, Jennifer A. Emond, Hannah Badland, Rodrigo Reis, Olga Sarmiento, Jordan Carlson, James F. Sallis, Ester Cerin, Kelli Cain, Terry Conway, Grant Schofield, Duncan J. Macfarlane, Lars B. Christiansen, Delfien Van Dyck, Rachel Davey, Ines Aguinaga-Ontoso, Deborah Salvo, Takemi Sugiyama, Neville Owen, Josef Mitáš, Loki Natarajan

**Affiliations:** 1 Division of Behavioral Medicine, University of California, San Diego, La Jolla, California, USA; 2 Community and Family Medicine, Dartmouth College, Hanover, New Hampshire, USA; 3 McCaughey VicHealth Centre for Community Wellbeing, University of Melbourne, Melbourne, Victoria, Australia; 4 Pontiff Catholic University of Paraná, Curitiba, Brazil; 5 Faculty of Medicine, Universidad de los Andes, Bogotá, Colombia; 6 Centre for Physical Activity and Nutrition Research, Deakin University, Melbourne, Victoria, Australia; 7 Institute of Human Performance, University of Hong Kong, Pokfulam, Hong Kong; 8 Human Potential Centre, Auckland University of Technology, Auckland, New Zealand; 9 Department of Sports Science and Clinical Biomechanics, University of Southern Denmark, Odense, Denmark; 10 Department of Movement and Sport Sciences, Ghent University, Ghent, Belgium; 11 Centre for Research and Action in Public Health, University of Canberra, Canberra, Australian Capital Territory, Australia; 12 Department of Health Sciences, Public University of Navarra, Pamplona, Spain; 13 Nutrition and Health Research Center, Instituto Nacional de Salud Publica (National Institute of Public Health), Cuernavaca, Morelos, Mexico; 14 Michael & Susan Dell Center for Healthy Living, University of Texas Health Science Center at Houston, School of Public Health (Austin Regional Campus), Austin, Texas, USA; 15 Nutrition and Health Sciences, Graduate Division of Biological and Biomedical Sciences, Emory University, Atlanta, Georgia, USA; 16 School of Population Health, University of South Australia, Adelaide, South Australia, Australia; 17 Behavioural Epidemiology Laboratory, Baker IDI Heart and Diabetes Institute, Melbourne, Victoria, Australia; 18 Institute of Active Lifestyle, Faculty of Physical Culture, Palacký University, Olomouc, Czech Republic

## Abstract

**Introduction:**

Prevalence of walking and cycling for transport is low and varies greatly across countries. Few studies have examined neighborhood perceptions related to walking and cycling for transport in different countries. Therefore, it is challenging to prioritize appropriate built-environment interventions.

**Objectives:**

The aim of this study was to examine the strength and shape of the relationship between adults’ neighborhood perceptions and walking and cycling for transport across diverse environments.

**Methods:**

As part of the International Physical activity and Environment Network (IPEN) adult project, self-reported data were taken from 13,745 adults (18–65 years) living in physically and socially diverse neighborhoods in 17 cities across 12 countries. Neighborhood perceptions were measured using the Neighborhood Environment Walkability Scale, and walking and cycling for transport were measured using the International Physical Activity Questionnaire–Long Form. Generalized additive mixed models were used to model walking or cycling for transport during the last seven days with neighborhood perceptions. Interactions by city were explored.

**Results:**

Walking-for-transport outcomes were significantly associated with perceived residential density, land use mix–access, street connectivity, aesthetics, and safety. Any cycling for transport was significantly related to perceived land use mix–access, street connectivity, infrastructure, aesthetics, safety, and perceived distance to destinations. Between-city differences existed for some attributes in relation to walking or cycling for transport.

**Conclusions:**

Many perceived environmental attributes supported both cycling and walking; however, highly walkable environments may not support cycling for transport. People appear to walk for transport despite safety concerns. These findings can guide the implementation of global health strategies.

**Citation:**

Kerr J, Emond JA, Badland H, Reis R, Sarmiento O, Carlson J, Sallis JF, Cerin E, Cain K, Conway T, Schofield G, Macfarlane DJ, Christiansen LB, Van Dyck D, Davey R, Aguinaga-Ontoso I, Salvo D, Sugiyama T, Owen N, Mitáš J, Natarajan L. 2016. Perceived neighborhood environmental attributes associated with walking and cycling for transport among adult residents of 17 cities in 12 countries: the IPEN study. Environ Health Perspect 124:290–298; http://dx.doi.org/10.1289/ehp.1409466

## Introduction

The 2011 *United Nations High-level Meeting on Non-Communicable Diseases* identified increasing physical activity as one of five priority intervention areas to reduce the impact of noncommunicable diseases, noting modification of the built environment to support habitual physical activity as a key focus area (Beaglehole et al. 2011). Engaging in active transport (AT) (i.e., walking and cycling for travel purposes) provides opportunities to habitually accumulate physical activity (Badland and Schofield 2008), and those who engage in AT tend to be more active in duration and frequency than those who do not (Berrigan et al. 2006).

People who walk and cycle for transport have been reported to be less likely to be overweight or obese than those who travel by private motor vehicle (Badland and Schofield 2008; Bassett et al. 2008). Additional benefits of AT reported by previous studies include greater social inclusion (Currie et al. 2007), improved air quality, and reduced traffic congestion, vehicle miles traveled, and road infrastructure expenditure (Haines et al. 2009). The prevalence of walking and bicycling for transport varies worldwide, with estimated bicycling rates ranging from 1–2% in North America and Australasia to 25% in The Netherlands (Bassett et al. 2008; González et al. 2014; Merom et al. 2010; Reis et al. 2013). Because private motor vehicle journeys often cover distances (< 5 km) that are feasible for AT modes, there is great potential to replace automobile trips with AT that provides health benefits (Dekoster and Schollaert 1999; Keller 2004; Sugiyama et al. 2012).

The role of environmental and policy strategies to increase AT has recently received attention, with calls for further evidence on the most relevant and potentially modifiable environmental attributes (Fraser and Lock 2011). Several studies have explored associations between built-environment attributes and walking or cycling for transport (Badland et al. 2008; Pucher and Buehler 2008; Saelens and Handy 2008). Few of these studies, however, had sufficient power and variability to assess walking and cycling separately.

We hypothesize that identifying environmental attributes that benefit both modes of AT will be important to maximize health, social, and environmental gains in a fiscally constrained global environment. AT studies thus far have been primarily limited to Australasia, Europe, and North America (Bassett et al. 2008), and associations have been weak or inconsistent, possibly due to limited variability in the samples. Although objective measures of the built environment are important, perceptions of environments are also related to behavior and may provide complementary information. Some attributes, such as aesthetics, cannot be measured objectively; other attributes, such as sidewalks, are simply unavailable as objective data in most cities. International studies performed using comparable methods can identify the relevant differences and similarities between countries and inform evidence-based international and country-specific interventions to increase AT.

The purpose of the present study, conducted across diverse cities and countries, was to examine the strength and shape of the relationship of adults’ perceptions of several built-environment attributes, selected for *a priori* theoretical and empirical reasons, with walking and cycling for transport. Analyses controlled for multiple potential confounding variables and interactions by city were explored to assess the international generalizability of the findings. Understanding these relationships is critical for guiding policy and practice to support walking and cycling for transport.

## Methods

*Study design and locations.* The International Physical activity and Environment Network (IPEN) adult study is an observational epidemiologic multicountry cross-sectional study using a common design and comparable methods (Kerr et al. 2013). Seventeen cities from 12 countries participated in the study: Australia (AUS): Adelaide; Belgium (BEL): Ghent; Brazil (BR): Curitiba; China (CN): Hong Kong; Colombia (COL): Bogota; Czech Republic (CZ): Olomouc and Hradec Kralove; Denmark (DEN): Aarhus; Mexico (MEX): Cuernavaca; New Zealand (NZ): North Shore, Waitakere, Wellington, and Christchurch; Spain (SP): Pamplona; the United Kingdom (UK): Stoke-on-Trent; and the United States (US): Seattle, Washington, and Baltimore, Maryland. In each of the 17 cities, study neighborhoods were chosen first, and then participants were recruited from these neighborhoods (Frank et al. 2010; Kerr et al. 2013).

Neighborhood selection. Neighborhoods were chosen in each city to maximize variability in environmental attributes and socioeconomic status (SES). Neighborhood walkability index (Frank et al. 2010) determined objectively with geographic information systems (GIS) was used for this purpose, except in Spain, where neighborhoods were selected based on their construction date (a proxy measure of walkability) (Berrigan and Troiano 2002). For each country, we used the smallest administrative area unit that represented a neighborhood-level geographic sector for the development of the walkability measures (Adams et al. 2014).

Administrative units were ranked in deciles based on the normalized walkability index and on neighborhood-level SES data drawn from the census (e.g., household income, education attainment, or ethnicity) in each city. The walkability index and census-based SES scores were crossed to produce four neighborhood quadrants: high walkability/high SES; high walkability/low SES; low walkability/high SES; and low walkability/low SES (Kerr et al. 2013). Equal numbers of neighborhoods were selected from each of the quadrants. The neighborhood selection methods for each country have been described elsewhere (Adams et al. 2014; Kerr et al. 2013).

*Participant recruitment.* Participants were systematically selected using addresses from the identified neighborhoods. Four countries recruited and conducted data collection by phone and mail, and the remaining eight countries contacted households in person. Adults living in the selected neighborhoods were contacted and invited to complete surveys on their physical activity behaviors and perceptions of the neighborhood environment. Study dates ranged from 2002 to 2011, with participants’ ages ranging between 16 and 94 years. Analyses were performed on participants 18–66 years old because only three countries had a wider age range. Six countries used monetary incentives, three countries provided nonmonetary incentives (e.g., physical activity feedback), and three countries provided no incentives for recruitment. Participants were recruited across the seasons to control for variations in weather that may have affected physical activity. Further details on the participant recruitment response rates across countries are available elsewhere (Kerr et al. 2013).

*Quality control and comparability.* All investigators completed the San Diego State University Institutional Review Board training (because the grant was housed at this institution during the data collection phase) and satisfied the NIH Fogarty International Center ethics requirements and their own research institution’s ethics requirements. All participants provided signed informed consent for participation in their home country. Participant confidentiality for pooled data was maintained by deidentification using numeric identification codes rather than names.

All survey data were assessed for completeness by sites and were double-checked by the single coordinating center at the University of California, San Diego. Study investigators in each country provided back-translations of surveys, and the comparability of item wording, response options, and number of items was assessed by two independent raters who were experts in the area (B. Saelens, University of Washington; B. Ainsworth, Arizona State University). Only comparable items were included in the scales created and employed in the current analyses.

*Measures.* Physical activity. The self-administered International Physical Activity Questionnaire long form (IPAQ–LF) was used to measure participants’ physical activity for recreation and transport purposes. The IPAQ–LF assesses the frequency and duration of activities separately across multiple domains (i.e., recreation, transport, occupation, household) (Craig et al. 2003). The IPAQ–LF has been evaluated in 14 studies across 12 countries on five continents and has been found to have acceptable test–retest reliability (0.8). Validity was tested by correlations with accelerometers (0.3), and the results were comparable to those of other self-report surveys (Craig et al. 2003). Seven countries collected IPAQ–LF data using interview techniques, and three countries provided an online version in addition to, or instead of, mailing out paper copies.

The IPAQ–LF items used in the present analyses assessed walking and cycling for transport. The items queried the number of days during the last week that were spent walking or cycling for ≥ 10 min to get from place to place and the usual minutes spent doing so per day. Total minutes per week spent walking or cycling for transport (days × minutes per day) were calculated and treated as continuous variables. In addition, dichotomous outcome measures were derived to represent any walking or cycling for transport during the last week (no, yes) that lasted for ≥ 10 min, and whether ≥ 150 min of walking or cycling for transport during the last week was accumulated (no, yes). This total reflects the current international adult physical activity guidelines [World Health Organization (WHO) Centre for Health Development 2011].

Perceived environment. Many studies have established the independent predictive value of resident perceptions of the neighborhood environment, in addition to the objective assessment of neighborhood attributes (e.g., those based on audits or GIS), as they relate to physical activity (Adams et al. 2009; Gebel et al. 2009; Mason et al. 2013; Saelens and Handy 2008). Perceptions of neighborhood attributes were assessed among U.S. participants using the 67-item Neighborhood Environment Walkability Scale (NEWS), and perceptions among participants in the 10 remaining countries were assessed using original or slightly modified items from the NEWS scale in combination with items from the NEWS–A scale, an empirically derived abbreviated (54-item) version of the NEWS (Cerin et al. 2006). See Appendix for a list of common items employed in each city to assess each of the following environmental predictor subscales: neighborhood residential density, land use mix–access, street connectivity, pedestrian infrastructure, aesthetics, traffic safety, and crime safety. Subscale scores ranged from 0 to 1,044 for residential density, and from 1 (strongly disagree) to 4 (strongly agree) for all other items, with higher scores indicating more favorable environments (Cerin et al. 2013). In addition, we assessed perceived distances to walk to 13 common neighborhood destinations (also known as mixed-use diversity). Response options for each destination type were 1 = > 31-min walk or don’t know; 2 = 21- to 30-min walk; 3 = 11- to 20-min walk; 4 = 6- to 10-min walk; 5 = 1- to 5-min walk. The responses were averaged across the 13 destinations to create a score that ranged from 1 to 5, where higher values represented more destinations within a close walking distance. The reliability and validity of the NEWS and the NEWS–A have been documented in several countries (Cerin et al. 2007; De Bourdeaudhuij et al. 2003; Leslie et al. 2005; Malavasi et al. 2007), with all included scales having test–retest reliability interclass correlation coefficients (ICCs) > 0.75.

*Demographic variables.* Demographic items collected by all countries included age, sex, education, and marital status. Although the types of education available varied by country, education data from all countries could be categorized into college graduate or not. Marital status was recoded to indicate “married or living with a partner” versus not.

*Data analytic plan.* Descriptive statistics (means, medians, standard deviations, percentages, and percentages of missing values) were computed, as appropriate, by study city for all relevant variables. Data on at least one of the examined variables were missing for > 8% of the participants. To avoid potential biases associated with a complete-case analysis (Rubin 1987) and to improve efficiency, we used multiple imputation methods to impute missing values. Consequently, 10 imputed data sets were created for the main regression analyses (see below), as recommended (Rubin 1987; van Buuren 2012). Multiple imputations were performed using Markov chain Monte Carlo methods (Schafer 1997) to account for within-site administrative-unit-level cluster effects arising from the two-stage stratified sampling strategy employed at each study site. The 10 imputed data sets were created in R (R Core Team 2013) using the “mix” package for multiple imputation of mixed categorical and continuous variables, and following the model-building and diagnostic procedures outlined by van Buuren (2012). Nonimputed results yielded the same conclusions (data not shown). The main aims of this study were to estimate the strength and shape of associations of multiple perceived environmental attributes with walking and cycling for transport for the whole sample and to examine whether these associations varied by city. The built-environment variables we focused on had theoretical and empirical support for their inclusion (Saelens and Handy 2008).

Six physical activity outcomes were explored: any bouts (> 10 min) of walking or cycling (dichotomous), walking or cycling for ≥ 150 min per week (dichotomous), and total minutes of walking or cycling in those who walked/cycled for transport (continuous). The three different types of outcomes (any, ≥ 150 min, and total minutes) were explored because they have different implications for public health. For example, ≥ 150 min represents the amount suggested by international physical activity guidelines (WHO Centre for Health Development 2011); yet even small amounts of activity may be beneficial for health (Blair et al. 2004). Furthermore, examining total minutes spent walking or cycling allows the investigation of which built-environmental correlates are related to more walking/cycling among those who do any amount of these activities.

Generalized additive mixed models (GAMMs) were used for these analyses (Wood 2006). GAMMs can model data following various distributional assumptions (e.g., positively skewed physical activity data), account for dependency in error terms due to clustering (observations sampled from selected administrative units), and estimate complex dose–response relationships of unknown form (Wood 2006). In our analysis, the shape of dose–repose relationships was estimated using thin-plate splines (Wood 2006). Random intercepts were specified to account for within-administrative unit correlations. The appropriateness of the GAMMs and their link functions was assessed via residual plots; quasi-Akaike Information Criterion (qAIC) values were used for model selection (e.g., linear vs. nonlinear), whereby a lower qAIC was indicative of a better-fitting model (Wood 2006). For the current analysis, absolute differences in qAIC values ≥ 10 were used as the criteria for model selection (Burnham and Anderson 1998). For the dichotomous outcome variables, the GAMMs used binomial variance and logit link functions. The reported antilogarithms of the regression coefficient estimates of these models represented odds ratios of walking versus not walking, cycling versus not cycling, and meeting or not meeting the ≥ 150 min per week activity recommendations. For the “total minutes of walking” (or cycling) outcomes, we used a negative binomial regression model for overdispersed count data. The antilogarithm of the coefficients from the negative binomial models can be used to estimate proportional increases (or decreases) in minutes of walking (or cycling) associated with changes in environmental attributes.

A first set of models estimated the dose–response relationships of the perceived environmental attributes relevant to walking and cycling for transport with the outcomes, adjusting for study city, sociodemographic covariates, and design variables including neighborhood-level and SES. Separate models were run to estimate main associations of each environmental attribute. Quasi-AIC criteria were used to choose *a*) between curvilinear (thin-plate splines) and linear relationships of environmental attributes with outcomes, and *b*) whether to include two-way city by environmental attributes interaction effect estimates. Interactions were tested for each model to see whether there were significant (AIC < 10) differences in the relationships across the 17 cities. For variables where a significant interaction was found, the main associations for each city are presented as forest plots. For significant nonlinear associations, the shape of the curve is plotted. All analyses were performed in R (R Core Team 2013) using the “mix” (by J.L. Schafer in 2013: Estimation/Multiple Imputation for Mixed Categorical and Continuous Data; http://rpackages.ianhowson.com/cran/mix/), and “mgcv” (Wood 2006) packages.

## Results

*Descriptive results.* Table 1 describes the sample in each city. The total sample size was 13,745 adults. The study aimed to balance samples by walkability, SES, and sex, and the percentages demonstrate that these goals were achieved. Percentages of participants with a partner ranged from 44.8 to 74.2%, and percentages of particpants with a university degree ranged from 14.1 to 67.6%. The average age ranged from 34.0 to 46.6 years.

**Table 1:** Demographic characteristics of sample by city.

City	*n*	Low walk NH (%)	Low SES NH (%)	Female (%)	With partner (%)	College graduate (%)	Age (years) (mean ± SD)
AUS: Adelaide	2,650	51.4	47.9	64.0	56.5	46.3	44.5 ± 12.3
BEL: Ghent	1,166	50.0	49.7	52.1	73.4	60.9	42.7 ± 12.6
BR: Curitiba	697	49.8	50.2	52.9	58.1	38.7	41.1 ± 13.2
CN: Hong Kong	493	47.1	48.7	58.9	59.0	40.0	42.8 ± 11.7
COL: Bogota	963	44.8	59.5	63.7	53.4	22.2	40.0 ± 13.7
CZ: Hradec Kralove	167	53.3	31.7	60.5	47.4	26.1	34.0 ± 13.1
CZ: Olomouc	330	32.1	40.6	62.7	58.4	32.2	37.9 ± 14.7
DEN: Aarhus	642	46.6	43.9	56.7	65.4	48.0	39.0 ± 13.9
MEX: Cuernavaca	677	50.5	49.8	55.4	64.7	27.6	42.1 ± 12.6
NZ: Christchurch	495	50.3	50.3	55.8	55.4	32.0	41.7 ± 12.6
NZ: North Shore	511	50.3	33.3	63.9	70.4	38.3	41.1 ± 11.8
NZ: Waitakere	512	48.6	59.0	60.7	74.2	30.7	40.8 ± 11.8
NZ: Wellington	496	49.4	50.0	51.2	56.7	52.2	39.2 ± 12.7
SP: Pamplona	904	32.0	56.9	55.2	53.0	57.8	38.7 ± 14.2
UK: Stoke-on-Trent	843	77.5	47.1	56.1	44.8	14.1	43.0 ± 13.3
US: Baltimore	912	50.8	47.5	52.3	60.5	67.6	46.6 ± 10.7
US: Seattle	1,287	49.4	48.7	45.2	63.2	63.2	44.0 ± 11.0
Abbreviations: AUS, Australia; BEL, Belgium; BR, Brazil; CN, China; COL, Colombia; CZ, Czech Republic; DEN, Denmark; MEX, Mexico; NH, neighborhood; NZ, New Zealand; SD, standard deviation; SES, socioeconomic status; SP, Spain; UK, United Kingdom; US, United States; walk, walkability.							

Table 2 shows the varying range in the six AT outcomes across the 17 cities. The highest percentages reporting any walking for transport in the previous 7 days were found in Pamplona (SP) (92.3%), followed by Cuernavaca (MEX), and Bogota (COL) (90.3%); in addition, > 80% reported any walking for transport in Aarhus (DEN), the cities from the Czech Republic, and Wellington (NZ). Levels of any cycling for transport in the last 7 days were much lower (in contrast to any walking), ranging from 1.2% in Cuernavaca (MEX) to 62.5% in Aarhus (DEN).

**Table 2:** Prevalence of walking and cycling for transport outcomes by city assessed in previous week by IPAQ–LF.

City	Any walking for transport [*n* (%)]	Any cycling for transport [*n* (%)]	≥ 150 min walking for transport [*n* (%)]	≥ 150 min cycling for transport [*n* (%)]	Total minutes walking for transport (mean ± SD)^*a*^	Total minutes cycling for transport (mean ± SD)^*b*^
AUS: Adelaide	1,998 (77.1)	304 (11.8)	973 (36.7)	130 (4.9)	200.2 ± 412.7	27.7 ± 174.0
BEL: Ghent	608 (52.1)	504 (43.2)	194 (16.6)	194 (16.6)	79.3 ± 155.9	63.9 ± 119.7
BR: Curitiba	538 (77.3)	52 (7.5)	195 (27.9)	19 (2.7)	153.3 ± 300.0	14.7 ± 78.2
CN: Hong Kong	377 (78.9)	44 (9.7)	249 (50.5)	22 (4.4)	288.9 ± 881.2	18.8 ± 83.1
COL: Bogota	870 (90.3)	89 (9.2)	490 (50.9)	39 (4.1)	303.7 ± 490.9	21.7 ± 125.0
CZ: Hradec Kralove	139 (83.2)	59 (35.3)	87 (52.1)	26 (15.6)	298.1 ± 411.1	85.3 ± 233.7
CZ: Olomouc	272 (83.2)	60 (18.2)	203 (61.5)	24 (7.3)	401.5 ± 591.9	35.7 ± 141.0
DEN: Aarhus	514 (86.1)	401 (62.5)	229 (35.7)	188 (29.3)	190.8 ± 331.4	136.1 ± 222.5
MEX: Cuernavaca	611 (90.3)	8 (1.2)	303 (44.7)	1 (0.2)	325.7 ± 584.9	0.8 ± 8.5
NZ: Christchurch	278 (56.2)	66 (13.3)	84 (17.0)	29 (5.9)	79.7 ± 177.4	26.4 ± 110.5
NZ: North Shore	334 (65.5)	31 (6.1)	98 (19.1)	11 (2.2)	86.1 ± 154.8	14.1 ± 127.3
NZ: Waitakere	319 (62.3)	37 (7.2)	70 (13.7)	13 (2.5)	88.7 ± 279.8	12.6 ± 81.0
NZ: Wellington	422 (85.1)	40 (8.1)	210 (42.3)	20 (4.0)	180.0 ± 220.5	18.6 ± 128.0
SP: Pamplona	810 (92.3)	111 (12.4)	560 (61.9)	42 (4.6)	322.0 ± 353.1	23.1 ± 108.4
UK: Stoke-on-Trent	553 (65.8)	35 (4.2)	287 (34.0)	25 (3.0)	218.4 ± 426.3	13.9 ± 109.1
US: Baltimore	620 (68.1)	60 (6.6)	305 (33.4)	19 (2.1)	171.4 ± 302.8	8.5 ± 43.1
US: Seattle	877 (68.3)	116 (9.0)	405 (31.5)	49 (3.8)	173.9 ± 359.4	17.0 ± 99.2
Abbreviations: AUS, Australia; BEL, Belgium; BR, Brazil; CN, China; COL, Colombia; CZ, Czech Republic; DEN, Denmark; MEX, Mexico; NZ, New Zealand; SP, Spain; UK, United Kingdom; US, United States. ^***a***^Total minutes in those who reported any walking. ^***b***^Total minutes in those who reported any cycling.						

The self-reported built-environment perceptions for neighborhood attributes varied greatly across cities. Residential density scores ranged from 18.2 in Waitakere (NZ) to 439.7 in Hong Kong (CN) (see Supplemental Material, Table S1). The differences in means of the other environmental variables across the cities were relatively small, approximately 0.7 in the variables assessed with a 4-point scale. Larger between-city variability was found for crime safety, from 2.1 in Bogota (COL) to 3.5 in Pamplona (SP). Participants in some cities [e.g., Curitiba (BR)] reported high land use access (3.7) but low traffic safety (2.4).

*Results of regression analyses.* Estimated associations of perceived environment subscales with four of the six outcome variables (≥ 150 min walking, total minutes walking, any cycling, and total minutes cycling) are shown in Table 3. Associations with any walking for transport (data not shown) were very similar to associations with walking ≥ 150 min, which may be more relevant to health outcomes. The low prevalence of participants meeting the ≥ 150 min cycling for transport outcome led us to present the environmental correlates of any cycling only.

**Table 3:** Estimated associations between perceived environmental attributes and walking and cycling for transport assessed in the previous week by IPAQ–LF*^a^*.

Environmental attributes	≥ 150 min walking for transport (*n* = 13,745)		Total minutes walking for transport in those who reported any walking (*n* = 4,939)		Any cycling for transport (*n* = 13,745)		Total minutes cycling for transport in those who reported any cycling (*n* = 851)	
	OR (95% CI)	*p*‑Value	exp(β) (95% CI)	*p*‑Value	OR (95% CI)	*p*‑Value	exp(β) (95% CI)	*p*‑Value
Residential density	NA^*b*^		1.001 (1.000, 1.001)	< 0.001	NA^*b*^		1.00 (0.999, 1.001)	0.805
Land use mix–access	1.33 (1.24, 1.42)	< 0.001	1.08 (1.03, 1.14)	0.001	1.24 (1.13, 1.36)	< 0.001^*c*^	1.05 (0.95, 1.15)	0.359
Street connectivity	1.15 (1.09, 1.21)	< 0.001^*c*^	1.06 (1.02, 1.11)	0.003	1.14 (1.06, 1.22)	0.001	1.00 (0.92, 1.08)	0.945
Pedestrian infrastructure	1.12 (1.04, 1.21)	0.002	1.04 (0.98, 1.10)	0.193	1.22 (1.10, 1.36)	< 0.001^*c*^	0.94 (0.84, 1.05)	0.267
Aesthetics	1.19 (1.11, 1.27)	< 0.001	1.05 (1.00, 1.11)	0.032	1.15 (1.05, 1.26)	0.003	1.01 (0.92, 1.11)	0.814
Traffic safety	0.92 (0.86, 0.97)	0.005	0.93 (0.89, 0.98)	0.002	1.14 (1.05, 1.24)	0.001	0.91 (0.83, 0.99)	0.033
Crime safety	0.99 (0.93, 1.05)	0.667	0.94 (0.90, 0.99)	0.010	1.17 (1.07, 1.28)	0.001	0.87 (0.80, 0.95)	0.002
Distance to local destinations	1.19 (1.12, 1.27)	< 0.001^*c*^	1.05 (1.00, 1.10)	0.052	1.16 (1.06, 1.27)	0.001	1.07 (0.99, 1.17)	0.108
Abbreviations: CI, confidence interval; OR, odds ratio. ^***a***^All models adjusted for participant sociodemographics, site, and study design variables (neighborhood–area unit and socioeconomic status). ^***b***^Association significant but not linear. Shape of relationship presented in Figure 1. ^***c***^Significant interaction by city, see Figure 2.								

**Figure 1:**
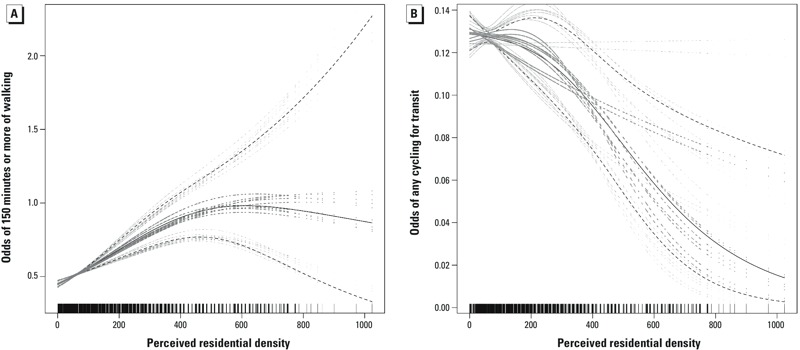
(*A*) Shape of significant nonlinear relationships between perceived residential density and ≥ 150 min walking for transport during the last week. (*B*) Shape of significant nonlinear relationships between perceived residential density and any cycling for transport during the last week. The solid lines represent point estimates [and dashed lines their 95% confidence intervals (CIs)]. The gray lines are the medians (and CIs) of the imputed point estimates. The tick marks above the *x*-axis represent the number of participants reporting this level of residential density. Residential density was the only variable with a significant nonlinear association.

**Figure 2:**
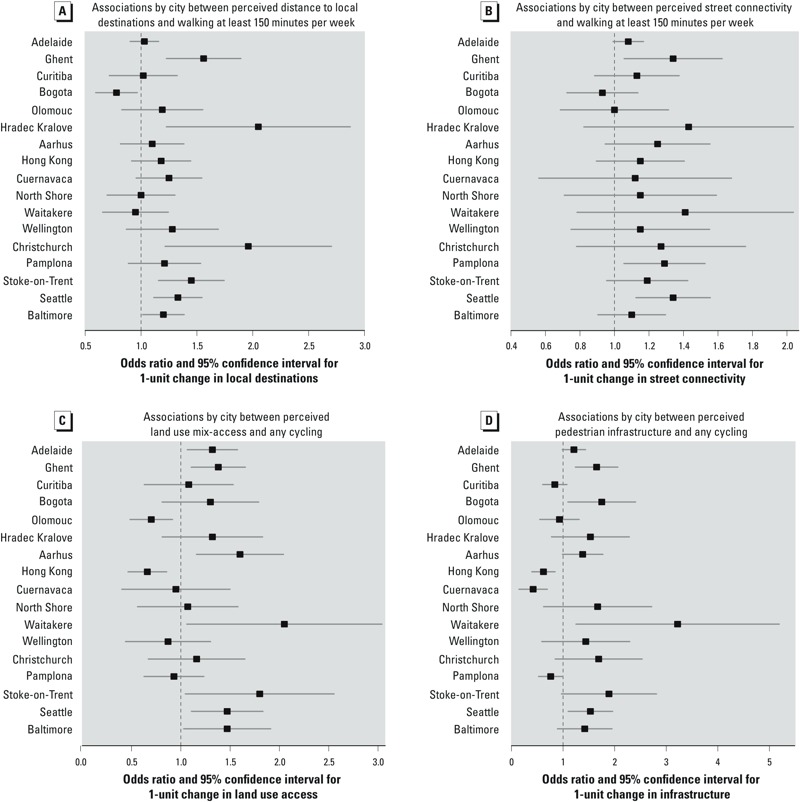
Forest plots of city-specific effects for any cycling for transport and ≥ 150 min walked for transport during the last week. The variables plotted demonstrated a significant interaction in the main analyses, highlighted by footnote c in Table 3. Interactions that were not significant are not plotted. Analyses adjusted for participant sociodemographics and study design variables (neighborhood–area unit and socioeconomic status).

Walking. There was a significant nonlinear association between perceived residential density and ≥ 150 min walking for transport that was positive up to a perceived density score of approximately 500, and flat or negative for higher scores (Figure 1A). Perceived land use mix–access, street connectivity, pedestrian infrastructure, aesthetics, and perceived distance to destinations all had significant positive linear associations with ≥ 150 min walking for transport during the previous week, whereas traffic safety had a significant negative association with this outcome (Table 3). In addition, there were significant differences among cities (interactions) for associations between this outcome and street connectivity and perceived distance to destinations (Figure 2A and B). Total minutes of walking for transport during the previous week was positively associated with perceived residential density, land use mix–access, street connectivity, and aesthetics, and was negatively associated with traffic and crime safety (Table 3).

Cycling. There was a significant nonlinear association between perceived residential density and any cycling for transport that was consistently negative in slope (Figure 1B). No other relationship was curvilinear. Any cycling for transport was positively associated with perceived land use mix–access, street connectivity, infrastructure, aesthetics, traffic safety, crime safety, and perceived distance to destinations when estimated across all populations (Table 3). However, there were significant interactions of perceived land use mix–access and infrastructure with study city, indicating significant variation among the different sites (Figure 2C and D).

## Discussion

These analyses explored the strength and shape of the relationship between perceived environment and walking/cycling for transport; city differences in these associations were also explored in this 17-city study. The variation in prevalence across cities for any walking for transport (52–92%) and cycling for transport (1–63%) demonstrates the value of studying such behaviors globally.

*Perceived residential density.* Perceived residential density had a significant nonlinear association with walking for transport ≥ 150 min during the previous week with a positive slope that subsequently plateaued, suggesting that walking for transport did not continue to increase with perceived density in extremely dense neighborhoods, for example, in neighborhoods with a perceived residential density score above approximately 500 [i.e., above the mean score for Hong Kong (439.7)]. Perceived residential density has consistently been positively associated with walking for transport in many other studies, but densities were not as varied nor as high as in the current study (Erikkson et al. 2012; Saelens and Handy 2008; Sugiyama et al. 2012; Van Holle et al. 2012; Witten et al. 2012).

In contrast, although the association between perceived density and any cycling for transport was also nonlinear, it was consistently negative across the entire range of perceived density scores, suggesting that increases in perceived density did not support cycling for transport. Previous studies, performed mostly with children, have found population density to be positively associated with cycling (Fraser and Lock 2011). Cycling also increased in adult participants who relocated to communities with higher residential density (Beenackers et al. 2012). Previous studies in Brazil, Sweden, and the United States, however, found that neighborhood walkability was not significantly related to cycling (Erikkson et al. 2012; Reis et al. 2013; Sallis et al. 2013). Because the lengths of cycling trips taken by adults typically exceed the 1-km neighborhood buffer used in such studies, it is not surprising that neighborhood features alone do not explain these behaviors. Residential density was negatively related to cycling in our study, possibly because highly connected pedestrian streets with crosswalks in densely populated areas do not support preferred cycling speeds. Bicycling rates in moderately dense cities, such as Aarhus, Denmark, are partly due to specialized infrastructure that supports uninterrupted cycling (Pucher and Buehler 2008). Examples of this specialized infrastucture include traffic lights timed to enhance cycling speeds and bikeways that are separate from any pedestrian or road traffic. Dense cities, such as Hong Kong, may not support cycling because distances to destinations are very short and can be covered by walking without the inconvenience of storing and transporting a bicycle in high-rise buildings. The present results may contradict results from previous studies conducted only in cities with limited variations in density. The present findings also demonstrate the importance of analyzing walking and cycling separately so that different environmental predictors can be detected.

*Perceived land use mix–access.* Perceived land use mix–access (having easy access to shops, recreational spaces, and transit stops) was significantly and positively associated with walking ≥ 150 min a week and total minutes of walking. This variable was also associated with any cycling for transport, but there was a significant city interaction. In two cities (Olomouc and Hong Kong), higher land use mix–access was significantly associated with lower odds of doing any cycling, although a positive association was found in seven cities (Van Holle et al. 2012). Stronegger et al. (2010) found that increased access to diverse land uses was related to walking for transport but not to cycling. Many previous studies of cycling found no associations for neighborhoods with multiple land uses and destinations, but these studies may have lacked statistical power (Van Holle et al. 2012). It is possible that the positive associations between land use access and both walking and cycling that were observed in our multicity study were evident in part because of the greater variability in exposures and outcomes than in previous study populations.

*Perceived street connectivity.* Studies that have reported on intersection density (an objective measure of perceived street connectivity), including studies performed in the United States, indicated that intersection density was associated with walking for transport (Saelens et al. 2012) but not with cycling (Sallis et al. 2013), potentially because of a lack of statistical power given the low cycling rates observed in the study sites. A European review (Van Holle et al. 2012) identified only one study (Van Dyck et al. 2011) that showed perceived street connectivity to be positively associated with cycling for transport but not with walking. In the present 17-city study, perceived street connectivity was related to both total minutes walked for transport and to any cycling for transport. The significant city interaction for obtaining ≥ 150 min of walking for transport revealed that street connectivity was positively associated with walking in all but 2 cities, but this association was significant in only 3 cities. This finding suggests that street connectivity alone may not be sufficient to support AT in most cities.

*Perceived pedestrian infrastructure.* We found that perceived infrastructure focused on facilities for pedestrians (i.e., street lights, crossings, and sidewalks) was positively related to ≥ 150 min of walking for transport. Sidewalks have been associated with walking for transport (not recreation) in some previous studies (McCormack et al. 2012; Sugiyama et al. 2012). In three cities, pedestrian infrastructure was negatively related to cycling for transport. The negative relationships between perceived infrastructure and any cycling in Pamplona, Hong Kong, and Cuernavaca suggest that these environments are designed predominantly for pedestrians and are less supportive of cycling (Mosquera et al. 2012).

*Perceived aesthetics.* In a recent European review, mixed results were observed for the association of aesthetics with AT behaviors (Van Holle et al. 2012), and previous studies have reported that aesthetics are related to recreational activity but not to AT engagement (Saelens and Handy 2008; Sugiyama et al. 2012; Witten et al. 2012).

However, aesthetics were found to be significant for both walking and cycling for transport in this 17-city study. Aesthetics ratings, like safety ratings, were low across all cities, suggesting that aesthetics may be an area for improvement with fewer cost implications than other structural changes (Becerra et al. 2013; Beenackers et al. 2012).

*Perceived traffic and crime safety.* We found that perceived safety from crime and traffic was positively associated with any cycling for transport but was negatively associated with the amount of walking/cycling among those who walked/bicycled. Safety from traffic was negatively associated with ≥ 150 min walked for transport per week. In contrast, previous studies did not find significant associations between perceived safety and walking (Van Holle et al. 2012). It may be that individuals walk regardless of safety issues because they have no other choice (e.g., they do not own a car, they must travel in areas not serviced by public transport). Furthermore, walkers may be more aware of threats to safety than those who do not walk (Adams et al. 2009).

Perceived safety is challenging to assess within an international context because participants rate their neighborhoods relative to their own experience, which can differ between countries. For example, a study performed in the city of New York, New York (USA), found that decreased homicide rates were related to increased population-level AT behaviors (Lovasi et al. 2013). Incorporating objective measures of safety (Foster and Giles-Corti 2008) and nuanced measures of bicycling risk may be required to improve our understanding of the likely complex relationship between perceived safety and physical activity.

*Perceived distance to local destinations.* We found that proximity of perceived destinations was marginally related to total walking for transport but was not associated with minutes spent cycling for transport. Perceiving local destinations to be nearby was significantly related to more adults doing any cycling for transport, but there was a significant city interaction for performing ≥ 150 min of walking for transport. The relationship between proximity of local destinations and walking was significant for six cities. Many other studies have shown associations between local destinations and walking (Saelens and Handy 2008). The negative relationship in Bogota (with the second-highest number of destinations) could be explained by trips made by walking being less than 10 min per day. The IPAQ–LF includes only trips with a duration of at least 10 mins; therefore, it might not capture shorter trips, which may be common in Bogota.

*Policy implications.* Important policy implications are indicated by the negative relationship between perceived residential density and cycling for transport and the two city interactions showing that walkable cities may not support cycling. In highly pedestrianized areas, it may be necessary to colocate cycling facilities. In some cities, it may be necessary to locate cyclists on a dedicated path on the road or in pedestrian centers, providing sidewalks with separate lanes for cyclists and pedestrians. In countries such as the United States, where walkability is limited but distances are cycleable, promotion of bicycle use may increase cycling rates. Pucher and Buehler (2008) concluded that substantial increases in cycling for transport require an integrated package of multiple complementary interventions, including provision of infrastructure and pro-bicycle programs, supportive land use planning, and restrictions on car use. The combination of economic, convenience, and health arguments in favor of cycling may be important to increase support for cycling investments among policy makers (Jones and Ogilvie 2012; Kahlmeier et al. 2010; Wooller et al. 2012). In some countries, such as Brazil, Mexico, and Colombia, cycling is considered a “poor man’s” mode of transport, but environmental support for cycling and changing social norms could alter such negative perceptions (Mosquera et al. 2012).

*Limitations.* The cross-sectional design of the present study limits evidence of causality that might support policy change. The variability and strength of the associations observed in this study, however, improve on those reported in previous cross-sectional studies and set the stage for a meaningful prospective study.

The present study focused only on self-reported perceptions of the built environment assessed with scales that had limited variability, even within this international context. Self-reported measures are limited when making international comparisons because people tend to make assessments relative to their own experience. Furthermore, thresholds from self-reported scales may not be helpful to policy makers. Some methodological differences across countries during the neighborhood selection and recruitment phases reflected local conditions and capacity and may have affected study findings and contributed to variations in associations across countries. However, the independent variables and outcome measures were collected consistently and were checked for comparability. The present analyses did not include assessment of cycling infrastructure because the NEWS was developed in the United States, where such infrastructure is mainly absent. A subset of countries deployed additional cycling infrastructure scales, and these will be explored in future analyses. Unfortunately, most cities do not have good GIS data for bicycling infrastructure, so future studies will need to employ street audits to assess the quality and quantity of these facilities.

The present study relied on self-reported measures of walking and cycling for transport. Total physical activity estimates are often overreported by the IPAQ–LF, but AT is usually more accurately reported than recreational physical activity (Johnson-Kozlow et al. 2006). Future studies should use GPS devices and/or travel diaries to identify trips in different modes (Carlson et al. 2015; Duncan et al. 2009). Finally, the IPAQ–LF elicits information about trips that take ≥ 10 min, potentially underestimating the relationship between the built environment and AT in highly walkable environments.

## Conclusions

This 17-city study of perceived environmental correlates of walking and cycling for transport demonstrated the importance of designing a study to capture environmental and behavioral variability. Many environmental attributes supporting both cycling and walking were found. People may walk for transport despite safety concerns. Highly walkable environments may not support cycling for transport. Our study highlights the importance of examining walking and cycling separately and of testing neighborhood attributes discretely.

## Supplemental Material

(181 KB) PDF

## Appendix. Neighborhood Environment Walkability Scale–Abbreviated (NEWS–A): IPEN Subscales and Items

*Residential density (weighted rating of housing types in neighborhood)* How common are…

Detached single-family residences
Townhouses or rows of 1–3-story houses
Apartments or condos with 1–3 stories
Apartments or condos with 4–6 stories
Apartments or condos with 7–12 stories
Apartments or condos with > 12 stories
Apartments or condos with > 20 stories

Land use mix–access

Stores are within easy walking distance of my home.
There are many places to go within easy walking distance of my home.
It is easy to walk to a transit stop (bus, train) from my home.

Street connectivity

The distance between intersections in my neighborhood is usually short (100 yards or less; the length of a football field or less).
There are many alternative routes for getting from place to place in my neighborhood (I don’t have to go the same way every time).

Pedestrian infrastructure

There are sidewalks on most of the streets in my neighborhood.
My neighborhood streets are well lit at night.
Walkers and bikers on the streets in my neighborhood can be easily seen by people in their homes.
There are crosswalks and pedestrian signals to help walkers cross busy streets in my neighborhood.

Aesthetics

There are trees along the streets in my neighborhood.
There are many interesting things to look at while walking in my neighborhood.
There are many attractive natural sights in my neighborhood (such as landscaping, views).
There are attractive buildings/homes in my neighborhood.

Traffic safety

There is so much traffic along nearby streets that it makes it difficult or unpleasant to walk in my neighborhood.
The speed of traffic on the street I live on is usually slow (30 mph/50 kph or less).
Most drivers exceed the posted speed limits while driving in my neighborhood.

Crime safety

There is a high crime rate in my neighborhood.
The crime rate in my neighborhood makes it unsafe to go on walks during the day.
The crime rate in my neighborhood makes it unsafe to go on walks at night.

*Perceived distance to local destinations.* About how long would it take to walk from your home to the nearest…

Supermarket
Other food/grocery, small grocery/convenience, fruit/veg market, bakery, butcher shop
Post office
Any school, elementary, other, nursery
Transit stop
Any restaurant, fast food, non–fast food, café/coffee place
Park/other public open space
Gym/fitness facility, recreation center, swimming pool
Library
Video store
Drug store/pharmacy
Bookstore
Other shops and services
