# Supplementary material for: Perceived Neighborhood Environmental Attributes Associated with Walking and Cycling for Transport among Adult Residents of 17 Cities in 12 Countries: The IPEN Study
Source: Environ Health Perspect. 2015 Jul 17;124(3):290–8. doi: 10.1289/ehp.1409466 (PMC4786986; doi:10.1289/ehp.1409466)
Supplement: (181 KB) PDF [file ehp.1409466.s001.acco.pdf]

Environ Health Perspect
DOI: 10.1289/ehp.1409466

Note to Readers: *EHP* strives to ensure that all journal content is accessible to all readers. However, some figures and Supplemental Material published in *EHP* articles may not conform to 508 standards due to the complexity of the information being presented. If you need assistance accessing journal content, please contact ehp508@niehs.nih.gov. Our staff will work with you to assess and meet your accessibility needs within 3 working days.

Supplemental Material

Perceived Neighborhood Environmental Attributes Associated with Walking and Cycling for Transport among Adult Residents of 17 Cities in 12 Countries: The IPEN Study

Jacqueline Kerr, Jennifer A. Emond, Hannah Badland, Rodrigo Reis, Olga Sarmiento, Jordan Carlson, James F. Sallis, Ester Cerin, Kelli Cain, Terry Conway, Grant Schofield, Duncan J. Macfarlane, Lars B. Christiansen, Delfien Van Dyck, Rachel Davey, Ines Aguinaga-Ontoso, Deborah Salvo, Takemi Sugiyama, Neville Owen, Josef Mitáš, and Loki Natarajan

Table of Contents

Table S1: Perceptions of the built environment by city (means ± standard deviation shown).

Table S1: Perceptions of the built environment by city (means ± standard deviation shown).

City	Residential density index	Land use access	Street connectivity	Infrastructure	Aesthetics	Traffic safety	Crime safety	13 local destinations
AUS: Adelaide	35.7 ± 40.6	3.5 ± 0.7	2.8 ± 0.9	3.0 ± 0.6	2.9 ± 0.7	2.8 ± 0.8	3.0 ± 0.8	3.9 ± 0.7
BEL: Ghent	83.8 ± 73.3	3.3 ± 0.6	2.7 ± 0.7	2.8 ± 0.5	2.5 ± 0.6	2.4 ± 0.6	3.1 ± 0.6	3.6 ± 0.9
BRZ: Curitiba	100.3 ± 123.3	3.7 ± 0.5	3.3 ± 0.7	2.8 ± 0.8	2.8 ± 0.8	2.4 ± 0.8	2.3 ± 0.5	4.1 ± 0.5
CN: Hong Kong	439.7 ± 235.2	3.2 ± 0.8	2.9 ± 0.8	3.3 ± 0.6	2.7 ± 0.7	2.4 ± 0.6	2.2 ± 1.0	4.0 ± 0.8
COL: Bogota	77.1 ± 82.0	3.4 ± 0.5	3.2 ± 0.5	2.8 ± 0.5	2.5 ± 0.6	2.5 ± 0.5	2.1 ± 0.7	4.3 ± 0.5
CZ: Hradec Kralove	91.6 ± 69.6	3.4 ± 0.7	2.9 ± 0.6	3.2 ± 0.5	2.5 ± 0.6	3.1 ± 0.5	3.4 ± 0.6	3.9 ± 0.7
CZ: Olomouc	90.9 ± 70.2	3.4 ± 0.7	3.0 ± 0.7	3.1 ± 0.5	2.4 ± 0.6	2.9 ± 0.6	3.2 ± 0.6	3.9 ± 0.6
DEN: Aarhus	86.1 ± 65.4	3.6 ± 0.6	3.0 ± 0.6	3.1 ± 0.5	2.7 ± 0.6	2.8 ± 0.5	3.3 ± 0.6	4.2 ± 0.6
MEX: Cuernavaca	38.4 ± 41.4	3.3 ± 0.5	2.9 ± 0.5	2.6 ± 0.4	2.6 ± 0.5	2.4 ± 0.5	2.2 ± 0.7	3.7 ± 0.6
NZ: Christchurch	21.8 ± 24.7	3.3 ± 0.5	3.0 ± 0.5	2.9 ± 0.4	2.8 ± 0.6	2.7 ± 0.5	2.9 ± 0.6	3.9 ± 0.6
NZ: North Shore	28.6 ± 46.9	3.2 ± 0.6	2.7 ± 0.5	2.8 ± 0.3	2.8 ± 0.5	2.6 ± 0.5	3.0 ± 0.5	3.8 ± 0.7
NZ: Waitakere	18.2 ± 26.5	3.1 ± 0.5	2.7 ± 0.4	2.8 ± 0.4	2.8 ± 0.5	2.6 ± 0.5	2.9 ± 0.4	3.6 ± 0.7
NZ: Wellington	49.2 ± 67.7	3.4 ± 0.5	2.8 ± 0.5	2.9 ± 0.4	2.8 ± 0.5	2.8 ± 0.4	3.1 ± 0.4	4.1 ± 0.6
SP: Pamplona	200.2 ± 103.8	3.7 ± 0.5	3.2 ± 0.7	3.3 ± 0.5	2.8 ± 0.7	2.4 ± 0.7	3.5 ± 0.6	4.6 ± 0.4
UK: Stoke-on-Trent	39.8 ± 40.7	3.3 ± 0.8	3.1 ± 0.7	3.1 ± 0.5	2.2 ± 0.8	2.5 ± 0.7	2.9 ± 0.8	3.6 ± 0.7
US: Baltimore	60.8 ± 79.4	3.0 ± 0.8	3.0 ± 0.8	3.1 ± 0.6	3.1 ± 0.6	2.7 ± 0.7	3.4 ± 0.7	3.6 ± 0.9
US: Seattle	39.3 ± 56.7	3.2 ± 0.8	3.0 ± 0.8	3.0 ± 0.6	3.1 ± 0.7	2.7 ± 0.7	3.4 ± 0.6	3.8 ± 0.8

Australia (AUS), Belgium (BEL), Brazil (BR), Colombia (COL), Czech Republic (CZ), Denmark(DEN), China (CN), Mexico (MEX), New Zealand (NZ), Spain (SP), the United Kingdom (UK) and the United States (US).
